# The Effectiveness of Modified Atkins Ketogenic Diet on Children with Intractable Epilepsy: A Pilot Study from Indonesia

**DOI:** 10.1155/2023/9222632

**Published:** 2023-12-29

**Authors:** Achmad Rafli, Setyo Handryastuti, Mulya Rahma Karyanti, Yoga Devaera, Cut Nurul Hafifah, Irawan Mangunatmadja, Muzal Kadim, Elisabeth Siti Herini, Lora Sri Nofi, Ariek Ratnawati, Suci Fitrianti

**Affiliations:** ^1^Department of Child Health Dr. Cipto Mangunkusumo Hospital-Faculty of Medicine Universitas Indonesia, Jakarta, Indonesia; ^2^Department of Child Health Dr. Sardjito Hospital-Faculty of Medicine Public Health and Nursing Universitas Gadjah Mada, Yogyakarta, Indonesia; ^3^Nutrition and Food Service Unit, Dr. Cipto Mangunkusumo Hospital, Jakarta, Indonesia

## Abstract

**Background:**

The ketogenic diet has recently been explored as a potential treatment approach for intractable epilepsy in children and has been applied in various parts of the world. The ketogenic diet is also effective for the treatment of mood disorders, especially for adolescent and young adults with epilepsy. The Modified Atkins Diet (MAD) is the less restrictive type of ketogenic diet with similar principles as the classic type. However, no study has been conducted to evaluate the use of MAD in children with severe epilepsy in Indonesia. This study aims to assess the effectiveness, tolerance, compliance, and the adverse effects of MAD in children with intractable epilepsy during a 6-month monitoring period.

**Methods:**

This is a pilot experimental study involving children aged 2–18 years old with intractable epilepsy at the Pediatric Neurology and the Pediatric Nutrition & Metabolic Diseases Clinics at the Dr. Cipto Mangunkusumo Hospital Jakarta between November 2021 and June 2022.

**Results:**

A total of 31 subjects met the inclusion criteria and received the MAD in the first month, followed by 13 (41.9%) subjects in the third month, and 9 (29%) subjects in the sixth month. The MAD reduced the seizure frequency by 50% (*p* = 0.144), 62% (*p* = 0.221), and 83.3% (*p* = 0.028) in the first, third, and sixth months, respectively. The most frequent adverse effects are vomiting and diarrhea. Noncompliance was observed in 18 (58.1%) subjects. A sample of the MAD food menu guidebook was developed to make it easier for parents to adhere to the diet.

**Conclusions:**

The MAD reduces the mean seizure frequency in children with intractable epilepsy in the first, third, and sixth months, with a statistical significance in the sixth month. A further randomized, controlled, and multicenter clinical trial with a larger sample size and longer observation period is required. This trial is registered with Protocol ID 20-10-1323.

## 1. Background

Epilepsy is still the most common neurological disorder in children, with estimated 10.5 million cases worldwide and 3.5 million new cases being reported annually, 40% of them being diagnosed in less than 18 years old, and more than 80% cases occur in developing countries [1–3]. The underlying mechanism of epilepsy is the overexcitation of nerves and repeated synaptic connections between the hypersynchronized neurons, creating a disturbance between inhibition and excitation equilibrium in one area or spread throughout the brain [4–7]. Approximately, 6–14% of epilepsy cases are difficult to be controlled with antiepileptic drugs [7, 8]. Intractable epilepsy is characterized by uncontrolled seizures despite being given at least two antiepileptic drugs (OAE) at maximum doses, and there are drug-related side effects that cannot be tolerated [1–4]. The classic ketogenic diet is a high-fat with restricted protein and carbohydrates diet that has risen as the mainstay dietary treatments of intractable or refractory epilepsy and has been gradually applied worldwide [5, 7, 9]. The understanding of the mechanism of action is incomplete, but the proposed theory propounds that the ketogenic diet mimics the fasting state, of which the glucose level in the blood is reduced, making the amount of ketone bodies (beta-hydroxybutyrate) as the byproduct of the diet are increased in the blood and cerebrospinal fluid, acting as the primary fuel of metabolism [9–12]. This state of catabolism modifies neuronal metabolism and excitability, and mitochondrial function and energy reserve, subsequently reducing the seizure frequency [12, 13]. However, despite its anticonvulsant effect, the loss of compliance over a period of time due to its strict nature and the adverse effects are deemed as the major problems of classic ketogenic diet, compromising its applicability in daily settings [5, 6, 10].

The Modified Atkins Diet (MAD) has risen as the alternate dietary treatment for intractable epilepsy [14, 15]. It is based on the same principle as the classic ketogenic diet but with nonrestricted calories and fluid intake, allowing patients to consume more fluid, protein, and carbohydrate [6, 14, 15]. The fat-protein and carbohydrate ratio in the MAD is 1 : 1, while the classic ketogenic diet is 3-4 : 1 [13–15]. With its less strict nature, MAD has more advantages than the classic ketogenic diet by being more feasible to be conducted in outpatient setting without the necessity to maintain a fasting state, making the adherence better its classic ketogenic counterpart [15–18]. Previous studies comparing the efficacy of the classic ketogenic diet and the MAD find that their overall efficacy in reducing seizures are comparable, except in children of 1–3 years of age [4, 6]. In children with retractable epilepsy under 3 years old, the classic ketogenic diet groups have been reported to have lesser baseline seizures frequency than the MAD, possibly due to its strict nature of inducing fasting state [13–16]. However, it has more adverse effects and a larger dropout rate than the MAD groups [4–6].

The MAD has been applied in some of the intractable epilepsy children at Dr. Cipto Mangunkusumo (RSCM), but without an established standard protocol. Although several foreign analyses have been published regarding the effectiveness of the MAD and classic ketogenic diet in children with severe epilepsy for 3–6 months, there has been no study evaluating the effectiveness of MAD in children with intractable epilepsy in Indonesia [4, 6, 18]. In addition, the MAD menu has not been widely available for children with epilepsy in Indonesia. This study aims to examine the effectiveness, tolerance, compliance, and adverse effects of the MAD in retractable epilepsy children within the six-month monitoring period. The findings of this study will contribute to the current knowledge that will serve as the basis its applicability in children with retractable epilepsy. The study hypothesis is that the MAD ketogenic diet can lower the frequency of seizures in children with intractable epilepsy, with minimal adverse effects.

## 2. Methods

**Figure 1:**
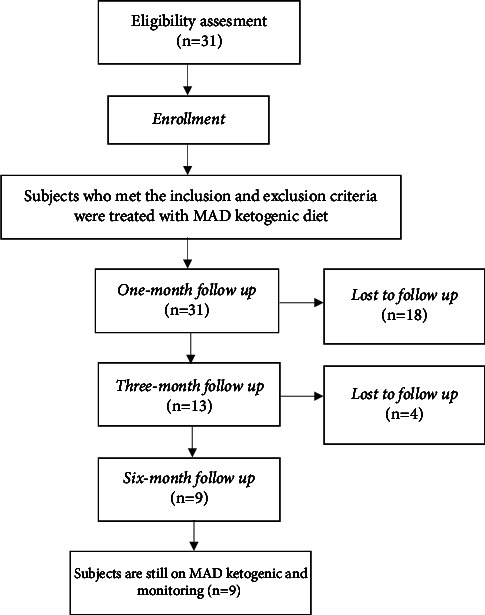
The flowchart of recruiting research subjects.

This is a pilot experimental, pre- and posttreatment clinical trial in children with intractable epilepsy conducted from November 2021 to June 2022 at the Pediatric Neurology Clinic, the Pediatric Nutrition and Metabolic Diseases Clinic, the Children's Nutrition Service Installation, and the Laboratory at RSCM. The inclusion criteria include children aged 2-18 years old with intractable epilepsy, well-nourished or with mild malnutrition, and who were able to consume solid food. The exclusion criteria are children with severe malnutrition, metabolic diseases (e.g., pyruvate carboxylase deficiency, primary carnitine deficiency, palmitoyltransferase deficiency I or II, and carnitine translocase deficiency), children with liver or kidney disorder, or food allergy, and refusal from parents or caregiver. The flowchart of subjects' recruitment, monitoring the MAD for first, third and sixth month, and the dropout rate is shown in Figure 1.

**Figure 2:**
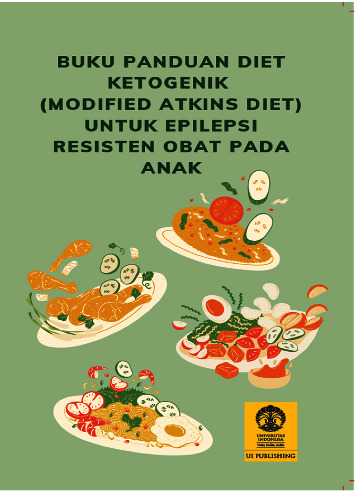
Guidebook and sample MAD ketogenic diet menu for children with intractable epilepsy in Indonesia.

This study uses the consecutive sampling method, with the total sample size being 31 subjects. The primary outcome is the reduction of seizure frequency before and after the administration of the MAD ketogenic diet. We make and use the book of seizure diary for children with intractable epilepsy to record frequency of seizure. This includes changes in the number of seizure frequencies in each subject before and after being given the ketogenic diet in 1, 3, and 6 months, where the average urine ketone level is achieved with a value of 0.5–16 mmol. The secondary outcomes are tolerance, compliance, and adverse effects of the MAD ketogenic diet. The menus of the MAD ketogenic diet were prepared and monitored throughout the study by the pediatric nutrition and metabolic diseases consultant and pediatric dietitians. We created a user-friendly guidebook of instructions and examples of the MAD food menu that are easy to adopt and inexpensive, with a familiar taste for Indonesian children (Figure 2).

Data were analyzed using the IBM Statistical Package for the Social Sciences (SPSS Statistics) version 25.0 software, using the Wilcoxon test. The ethics of this study was approved by the Health Research Ethics Committee of the Faculty of Medicine, University of Indonesia (No. KET.1135/UN2.F1/ETIK/PPM.00.02/2021).

## 3. Results

**Figure 3:**
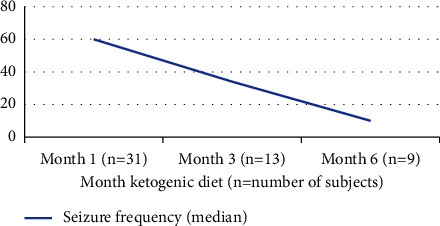
Seizure frequency (median) for 6 months on the MAD ketogenic diet.

Out of the 50 children with retractable epilepsy at RSCM Kiara, 31 children met the inclusion criteria as study subjects. The subjects followed the MAD ketogenic diet for 6 months and were evaluated in the first, third, and sixth month (Table 1). From the baseline 31 eligible subjects, there were dropouts throughout this study, subsequently leaving 13 (41.9%) subjects in the third month, and 9 (29%) subjects on the sixth month. This was due to several factors, such as poor compliance in 18 (58.1%) subjects during the COVID-19 pandemic, side effects of the diet in 2 (6.45%) subjects, and death in 9 (29%) subjects. The total frequency of seizures in the first, third, and sixth month of the study are shown in Figure 3.

Out of the 9 subjects who lasted until the sixth month, it is found that the frequency of seizures was reduced post-MAD (*p*=0.028) (Table 2). The MAD was further classified by three stages, i.e., stages I, II, and III that were consisted of 30%, 60%, and 90% fat, respectively, all with 10–20 grams of carbohydrates/day [19]. Out of the total 31 subjects, there were 10 (32.6%) subjects in MAD stage I, 5 (16.1%) subjects in stage II, and 16 (51.6%) subjects in stage III. Examination of urine ketones is imperative for subjects doing the MAD to evaluate the state of ketosis, and it is considered positive when the dipstick color is purple at 4–8 mmol, as shown in Table 3.

The expected urine ketone value ≥4 mmol is the cutoff point in achieving ketosis and was found in 28.6% subjects in stage II and stage III, and in 42.9% subjects in stage I of the MAD. During the MAD, several changes in lipid profile, impaired liver/kidney function, and increased uric acid levels occurred in our subjects, as shown in Table 4. The adverse effects of MAD ketogenic diet found in this study were vomiting (9.7%), diarrhea (6.5%), hyperactivity (3.2%), and electrolyte imbalance (3.2%) (Table 5). The examples of the MAD menu made by study subjects during the 6 months period are listed in Table 6.

## 4. Discussion

This is the first experimental study in Indonesia that evaluates the effect of the MAD in reducing frequency of seizures in children with intractable epilepsy. Our results reveal a significant reduction in seizure frequency at the end of the sixth month of intervention with MAD. Although the statistically significant result was found in the sixth month (83.3% of seizure reduction, *p*=0.028), a significant clinical reduction in seizures was observed since the first month of MAD. This is also an important finding because the decreased seizure frequency indicates successful epilepsy treatment, which improves the quality of life [17–20].

Our findings are comparable to those of previous studies by Kossoff et al. [19] and Kang et al. [21] that the administration of the MAD for 6 months in children with intractable epilepsy reduced the mean seizure frequency through the achievement of moderate ketosis [14, 19, 21]. These studies also find that more than 50% of their subjects who made through the end of the studies have achieved >50% reduction of seizures, with some had reached seizure-free state [14, 19, 21]. Kim et al. also reports similar findings, but by comparing the efficacy of the MAD with the classic ketogenic diet in children with intractable epilepsy [20]. This study finds that the classic ketogenic diet is more superior in reducing seizures, as the classic ketogenic group had a lower percentage of seizures than the MAD group. The difference, however, is not statistically significant [20]. Sharma et al. also evaluated the efficacy of the MAD in children with intractable epilepsy by comparing with their control group that received no dietary treatment [17]. This study finds that >50% reduction of seizures was higher in the MAD group [17]. A meta-analysis by Rezaei et al. supports the overall comparability of the MAD and classic ketogenic diet in reducing seizures who found that the MAD does not substantially differ from the classic ketogenic diet in ≥50% and ≥90% reduction of seizure in the third and sixth month after the initiation of both diets [22].

Urinary ketone was evaluated throughout the study. Positive urine ketone is defined as the urinary ketone level minimum of 4 mmol, and most of our subjects had achieved urinary ketosis in the first, third, and sixth months of evaluation. However, one of the drawbacks of evaluation urinary ketone is its potential fluctuating values [23–25]. The ideal ketosis monitoring is by blood examination, but the procedure is invasive for children and expensive [17, 26]. The extent of ketosis required to achieve the anticonvulsant effect in the MAD is still controversial [27, 28]. Neal et al. [13] argues that ketosis is not always proportionately correlated with lower seizure outcomes, but some studies suggest otherwise [19, 20, 23]. In most patients on the MAD, lower ketosis was not sufficient to control the intractable seizures, as previous studies show that patients who showed unfavorable outcomes had more fluctuating beta-hydroxybutirate levels in blood [23, 25]. There is also an association between higher urinary ketone levels and lower seizure frequencies [23, 25]. To achieve the state of ketosis, the modification of the Atkins diet was performed by reducing carbohydrate to 10–20 grams/day, similar with previous studies [19, 21, 28]. The MAD provided a more liberal consumption of carbohydrates and proteins, making it more applicable as a dietary therapy; however, it also poses a challenge to maintain the consistency of ketosis [23, 24, 29]. Hence, there is a necessity to monitor urinary ketosis throughout the study.

The MAD-related adverse effects found in this study were overall limited. The most common adverse effects found were gastrointestinal complaints of vomiting and diarrhea. In contrast to the reports in other studies, none of our subject experience weight loss or developed kidney stones. Although several subjects developed hypercholesterolemia, majority of subjects had their cholesterol levels within the normal limits throughout the study. Our findings are also similar to the adverse effects of the MAD being reported by previous studies, with an increased lipid profile and gastrointestinal disturbances as the most common adverse effects that mostly occur at the beginning of the studies and were transient or well-controlled by conservative management throughout the studies [17, 20, 26].

Our 9 subjects who made it through to the sixth month continued to apply the MAD until 6 to 12 months, due to their parents' satisfaction of the reduced seizure frequency since starting the diet. This was similar to the study by Weber et al. that 3 out of their 15 subjects continued the diet for 12 months with relatively good effectiveness in reducing seizure frequency [24]. The doses of AED were reduced gradually started in the third and sixth of MAD for 11.1% and 66.7% subjects, respectively. Reducing AED doses were carried out within 1 to 2 months, following the recommendations of The International Ketogenic Diet Study Group in discontinuing ketogenic diet and the dose of AED in children with severe epilepsy [30–32].

The main limitation of our study is the high dropout rate throughout the study, which may have influenced our results. The most common reason of dropout was poor compliance that was found in 18 of our subjects (58%), mainly due to the restrictions of the diet and to lesser extent, the complications of the diet, as was found by Kim et al. [20] and Kang et al. [21]. Our study was conducted during the intense lockdown of COVID-19 pandemic that may contribute to the high dropout rate of our subjects. In addition, the lack of comparison to a controlled group also poses as one of the methodical limitations in our interventional study.

## 5. Conclusion

In summary, the MAD is effective in reducing the seizure frequencies in children with intractable epilepsy. The striking similarity of results between our study and previous similar studies suggest that the MAD is an effective and well-tolerated dietary treatment for children with intractable epilepsy. Its comparable efficacy with the classic ketogenic diet and lesser restriction of the diet support its applicability in the daily practice, especially outpatient settings. However, this present study raises important concerns of the compliance issue. Although the MAD is not as strict as the classic ketogenic diet, it is still a relative strict diet that may be challenging to comply with. This study has produced a guidebook of the Indonesian menu of MAD in order to make it easier for patients and families to follow this diet. Although it is still being gradually improved over time, it will be useful for future clinical use and studies. For future study, a randomized and controlled clinical trial with larger number of subjects is necessary to yield better results and evidence.

## Figures and Tables

**Table 1:** Characteristics of research subjects.

Subject characteristics	Total *n* = 31 (%)
*Gender (%)*	
Male	14 (45.2)
Female	17 (54.8)
Seizure age (months), median	17 (6–69)
Age (months), median	105 (28–198)
*The amount of OAE taken before the diet, n (%)*	
2 drugs	3 (9.7)
3 drugs	22 (71.0)
4 drugs	4 (12.9)
>4 drugs	2 (6.5)
*Nutritional status, n (%)*	
Well	19 (61.3)
Not enough	12 (38.7)
*Associated disease, n (%)*	
Intellectual disability	17 (54.8)
Cerebral palsy	7 (21.6)
Impaired vision	1 (3.2)
Sleep/eating disturbances	2 (6.5)
Hyperactive/aggressive	1 (3.2)
Microcephaly	3 (9.7)
*MRI/CT scan results, n (%)*	
Normal	1 (3.2)
Abnormal	30 (96.8)
*EEG results, n (%)*	
Normal	7 (22.6)
Abnormal	24 (77.4)
*Seizure type, n (%)*	
Tonic	8 (25.8)
Clonic	1 (3.2)
Clonic tonic	13 (41.9)
Focal	4 (12.9)
Myoclonic	2 (6.5)
Atonic	3 (9.7)
Seizure frequency before ketogenic diet/month	120 (30–150)

**Table 2:** The effect of giving the MAD ketogenic diet to children with intractable epilepsy in reducing seizure frequency.

Variable	Pre median (range)	Post median (range)	*p* value
Seizure frequency			
0^th^ and 1^st^ month (*n* = 31)	120 (30–150)	60 (13–148)	0.144
0^th^ and 3^rd^ month (*n* = 13)	90 (33–150)	34 (10–151)	0.221
0^th^ and 6^th^ month (*n* = 9)	60 (6–195)	10 (5–157)	0.028

^*∗*^Wilcoxon test.

**Table 3:** Urinary ketones formed from urine dipstick examination during subjects undergoing the MAD ketogenic diet.

Urine ketones formed *n*(%)	End of month 1 *n* = 31 (%)	End of month 3 *n* = 13 (%)	End of month 6 *n* = 9 (%)
Ketone average			
≥4	29 (93.5)	11 (84.61)	7 (77.8)
<4	2 (6.45)	2 (15.39)	2 (22.2)

**Table 4:** Laboratory parameters during the MAD ketogenic diet.

Laboratory parameters, *n*(%)	End of month 1 *n* = 31 (%)	End of month 3 *n* = 13 (%)	End of month 6 *n* = 9 (%)
Within normal limits	30 (94.4)	7 (53.84)	5 (55.6)
Hypercholesterolemia (total cholesterol ≥ 250 mg/dL or triglycerides ≥ 150 mg/dL)	1 (5.6)	5 (38.46)	3 (33.3)
Increased uric acid (≥7 mg/dL)	0 (0.0)	1 (7.6)	1 (11.1)

**Table 5:** Side effects during subjects undergoing the MAD ketogenic diet.

Side effects while monitoring	*n*(%)
None	24 (77.4)
Gag	3 (9.7)
Hyperactive	1 (3.2)
Electrolyte balance	1 (3.2)
Diarrhea	2 (6.5)

**Table 6:** Samples of MAD ketogenic diet food menu.

No	Patient	Age	Gender	Calories (kcal)	Protein (g/day)	Carbohydrates (g/day)	Diet stage	Sample menu's breakfast	Sample menu's lunch	Sample menu's dinner
1	AGS	7	Male	1620	17	5–10	I	1 egg omelet, 2 small beef sausages, 2 tablespoons of rice, and 1 tablespoon of cooking oil	1 fried chicken egg , 10 scrambled quail eggs, 1 tablespoon butter, and 1 tablespoon mayonnaise	Saute chayote + 1 bowl of cabbage, 1 tablespoon butter, and 1 piece of watermelon
2	MR	16	Male	2520	50	5–10	II	2 fried eggs, 1 cup of stir-fried kale , and 3 tablespoons of oil	3 pieces of fried chicken 3 pieces, 1 cup of stir-fried kale, and 3(1/2) tablespoons of oil	2 tails of fried fish, 1 bowl soup, and 3(1/2) tablespoons of oil
3	BFA	13	Male	2300	28	5–10	III	3 pieces of fried milkfish, 1 fried egg, and 8 tablespoons of oil	4 pieces of fried chicken, 1 piece of fried tempeh, 2 tablespoons of mayonnaise, and 6 tablespoons of oil	Fried chicken 3 pieces, apple 1 piece, and 3 tablespoons of oil
4	JDN	15	Female	2000	35	5–10	I	1 small bowl of broccoli carrot chicken soup and 10 ml of butter	2 pieces of fried chicken, 1 egg omelet, 1 small bowl of carrot meatball soup, and 10 ml of butter	1 plate of fried shredded chicken , 1 small bowl of carrot meatball soup, and 5 ml of butter
5	PTR	12	Male	2400	22	5–10	II	2 fried eggs, 1 piece of fried chicken, 1 piece of tempeh, and 3 tablespoons of oil	4 pieces of fried chicken, 1 piece of tempeh, 3 (1/2) tablespoons of oil, and 1 bowl of spinach	3 pieces of fried chicken, 1 egg omelet, and 3 tablespoons of oil
6	AIP	3	Female	1000	18	10	III	2 fried eggs, 1 piece of tempeh, and 4 tablespoons of oil	1 bowl of fried shredded chicken and 6 tablespoons of oil	1 cup spinach, 1 cup shredded fried cobs, and 5 tablespoons of oil
7	SH	13	Male	2100	50	5	III	3 champ fried sausages, 1 tablespoon of full cream powdered milk, 4 kenzler meatballs, 3 tablespoons of mayonnaise, and 3 tablespoons of oil	Meatballs, 10 eggs, 1 glass of milk, 2 pieces of fried sausage, 13 tablespoons of oil, and 2 tablespoons of mayonnaise	4 kenzler meatballs, 2 cups milk, and 6 tablespoons of oil
8	VMR	6	Female	1500	30	10	III	2 pieces of fried chicken, 1 cup of spinach and carrots , 10 tablespoons of butter	2 pieces of fried chicken, 1 cup of spinach and carrots, and 10 tablespoons of butter	2 pieces of fried catfish, 1 cup of spinach and carrots, and 10 tablespoons of butter
9	ZZQ	6	Female	1600	32	10	II	1 grain of omelet and 2 tablespoons of oil	2 tablespoons of white rice, 2 eggs omelet, and 2 tablespoons of oil	1 piece of fried chicken, 1 tablespoon of mayonnaise, and 1 tablespoon of oil

## Data Availability

The SPSS data used to support the findings of this study are available from corresponding author upon request.
